# Best time to assess complete clinical response after chemoradiotherapy in squamous cell carcinoma of the anus (ACT II): a post-hoc analysis of randomised controlled phase 3 trial

**DOI:** 10.1016/S1470-2045(17)30071-2

**Published:** 2017-03

**Authors:** Robert Glynne-Jones, David Sebag-Montefiore, Helen M Meadows, David Cunningham, Rubina Begum, Fawzi Adab, Kim Benstead, Robert J Harte, Jill Stewart, Sandy Beare, Allan Hackshaw, Latha Kadalayil, ACT II study group

**Affiliations:** a Mount Vernon Centre for Cancer Treatment, Northwood, Leeds, UK; b University of Leeds, Leeds Cancer Centre, Leeds, UK; c Cancer Research UK and University College London Cancer Trials Centre, London, UK; d Royal Marsden Hospital, London, UK; e North Staffordshire Royal Infirmary, Stoke, UK; f Cheltenham General Hospital, Cheltenham, UK; g Belfast City Hospital Cancer Centre, Belfast, UK; h Northampton General Hospital, Northampton, UK; i Faculty of Natural and Environmental Sciences, University of Southampton, Southampton, UK

## Abstract

**Background:**

Guidelines for anal cancer recommend assessment of response at 6–12 weeks after starting treatment. Using data from the ACT II trial, we determined the optimum timepoint to assess clinical tumour response after chemoradiotherapy.

**Methods:**

The previously reported ACT II trial was a phase 3 randomised trial of patients of any age with newly diagnosed, histologically confirmed, squamous cell carcinoma of the anus without metastatic disease from 59 centres in the UK. We randomly assigned patients (by minimisation) to receive either intravenous mitomycin (one dose of 12 mg/m^2^ on day 1) or intravenous cisplatin (one dose of 60 mg/m^2^ on days 1 and 29), with intravenous fluorouracil (one dose of 1000 mg/m^2^ per day on days 1–4 and 29–32) and radiotherapy (50·4 Gy in 28 daily fractions); and also did a second randomisation after initial therapy to maintenance chemotherapy (fluorouracil and cisplatin) or no maintenance chemotherapy. The primary outcome was complete clinical response (the absence of primary and nodal tumour by clinical examination), in addition to overall survival and progression-free survival from time of randomisation. In this post-hoc analysis, we analysed complete clinical response at three timepoints: 11 weeks from the start of chemoradiotherapy (assessment 1), 18 weeks from the start of chemoradiotherapy (assessment 2), and 26 weeks from the start of chemoradiotherapy (assessment 3) as well as the overall and progression-free survival estimates of patients with complete clinical response or without complete clinical response at each assessment. We analysed both the overall trial population and a subgroup of patients who had attended each of the three assessments by modified intention-to-treat. This study is registered at controlled-trials.com, ISRCTN 26715889.

**Findings:**

We enrolled 940 patients from June 4, 2001, until Dec 16, 2008. Complete clinical response was achieved in 492 (52%) of 940 patients at assessment 1 (11 weeks), 665 (71%) of patients at assessment 2 (18 weeks), and 730 (78%) of patients at assessment 3 (26 weeks). 691 patients attended all three assessments and in this subgroup, complete clinical response was reported in 441 (64%) patients at assessment 1, 556 (80%) at assessment 2, and 590 (85%) at assessments 3. 151 (72%) of the 209 patients who had not had a complete clinical response at assessment 1 had a complete clinical response by assessment 3. In the overall trial population of 940 patients, 5 year overall survival in patients who had a clinical response at assessments 1, 2, 3 was 83% (95% CI 79–86), 84% (81–87), and 87% (84–89), respectively and was 72% (66–78), 59% (49–67), and 46% (37–55) for patients who did not have a complete clinical response at assessments 1, 2, 3, respectively. In the subgroup of 691 patients, 5 year overall survival in patients who had a clinical response at assessment 1, 2, 3 was 85% (81–88), 86% (82–88), and 87% (84–90), respectively, and was 75% (68–80), 61% (50–70), and 48% (36–58) for patients who did not have a complete clinical response at assessment 1, 2, 3, respectively. Similarly, progression-free survival in both the overall trial population and the subgroup was longer in patients who had a complete clinical response, compared with patients who did not have a complete clinical response, at all three assessments.

**Interpretation:**

Many patients who do not have a complete clinical response when assessed at 11 weeks after commencing chemoradiotherapy do in fact respond by 26 weeks, and the earlier assessment could lead to some patients having unnecessary surgery. Our data suggests that the optimum time for assessment of complete clinical response after chemoradiotherapy for patients with squamous cell carcinoma of the anus is 26 weeks from starting chemoradiotherapy. We suggest that guidelines should be revised to indicate that later assessment is acceptable.

**Funding:**

Cancer Research UK.

**Research in context**

**Evidence before this study**

Standard treatment for anal cancer is chemoradiotherapy. Guidelines previously recommended assessment of tumour response and biopsy at 6–12 weeks after starting treatment on the basis of several randomised trials and a population study. On the basis of this evidence salvage surgery was recommended to be done on patients with residual tumour shortly after completing chemoradiotherapy. However, present guidelines offer discordant advice on how often and when biopsy should be done and offer uncertainty over the optimum timing of response.

**Added value of this study**

Our post-hoc analysis of our trial data shows that tumour assessment at 26 weeks from the start of chemoradiotherapy is most strongly associated with progression and mortality compared with any earlier assessment. Many patients who do not have a complete clinical response at 11 weeks from the start of chemoradiotherapy do respond by 26 weeks and are therefore considered slow to respond to treatment.

**Implications of all the available evidence**

Present guidelines on the best timing of tumour response for anal cancer should be strengthened and an assessment of response at 26 weeks should be used in future treatment trials, and should be explored as a surrogate endpoint for survival and progression.

## Introduction

Standard treatment for anal cancer is chemoradiotherapy with concurrent fluorouracil and mitomycin.^1^, ^2^, ^3^, ^4^ Randomised phase 3 trials by the Radiotherapy Therapy Oncology Group (RTOG 98–11),^5^ the Action Clinique Coordonnées en Cancérologie Digestive (ACCORD 03) trial^6^ and the ACT II trial^7^ did not show benefit in terms of progression-free survival by increasing the radiotherapy boost dose,^6^ replacing mitomycin with cisplatin during chemoradiotherapy,^5^, ^6^ or by giving maintenance chemoradiotherapy after chemoradiotherapy.^7^

Guidelines for anal cancer recommend assessment of response at 6–12 weeks after starting treatment but discordance exists regarding consideration of early biopsy.^8^, ^9^, ^10^ Several randomised trials and one population study used a single response assessment as an early endpoint (4–8 weeks after the completion of trial treatments) and showed that 10–60% of patients did not respond to chemoradiotherapy.^1^, ^2^, ^3^, ^11^ On the basis of this evidence, salvage surgery can be done on patients who have residual tumour after completion of chemoradiotherapy.^3^, ^11^

**Figure 1:**
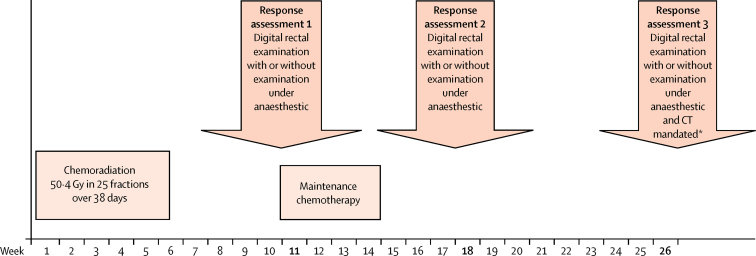
Treatment and assessment schedule in ACT II *Patients referred for surgical salvage as appropriate.

One of the primary endpoints of the ACT II trial was to assess whether cisplatin given concurrently with fluorouracil and radiotherapy produces a higher complete clinical response than mitomycin alone. A complete clinical response is defined as no evidence of residual tumour or nodal disease. Response was assessed at three timepoints up to 26 weeks from the start of chemoradiotherapy (figure 1). This post-hoc analysis of ACT II aimed to assess the difference in response at each of these timepoints and the association between having complete clinical response and progression-free or overall survival at each of these timepoints. This evidence can then be used indirectly to consider the best time for surgery. To the best of our knowledge, this assessment has not been prospectively investigated before.

## Methods

### Study design and participants

ACT II was a randomised phase 3 trial done in 59 centres in the UK and designed to investigate whether replacing mitomycin with cisplatin in the chemoradiotherapy schedule improves the complete response rate, and whether maintenance chemotherapy (fluorouracil and cisplatin) after chemoradiotherapy increases progression-free survival. Patients were randomised to one of four groups to receive mitomycin (12 mg/m^2^ on day 1) or cisplatin (60 mg/m^2^ on days 1 and 29) with fluorouracil (1000 mg/m^2^ per day on days 1–4 and 29–32) and radiotherapy (50·4 Gy in 28 day fractions); with or without two courses of maintenance chemotherapy (fluorouracil and cisplatin at weeks 11 and 14). The full trial methods and results have been reported previously.^7^

### Participants

Patients were eligible if they had newly diagnosed, histologically confirmed squamous cell carcinoma basaloid or cloacogenic carcinoma of the anal canal or margin, without metastatic disease, and considered fit for trial treatment; a glomerular filtration rate of 50 mL/min or more; acceptable haematological parameters (haemoglobin >100 g per L, platelets >100 × 10^9^ per L, white blood cells >3 × 10^9^ per L); liver function tests within twice normal range; and adequate cardiac function. There were no age limits. Exclusion criteria were other major malignancies likely to compromise life expectancy or completion of trial therapy, comorbidity including HIV-positive status and cardiac diseases, previous complete local excision, and previous radiotherapy to the pelvis. All patients provided written informed consent and the trial was approved by UK research ethics committees.

### Randomisation

Randomisation was done by minimisation and stratified by site, T and N stage, sex, age, and renal function. Allocation was concealed by use of a computer program (in the trial co-ordinating centre) to generate the treatment allocation. Site staff would telephone the trial co-ordingating centre, and the assigned treatment was provided for the next patient. Patients, clinicians (including those assessing patients) and investigators analysing data were not masked to treatment allocation.

### Procedures

Briefly, all patients received 50·4 Gy delivered in 28 daily fractions over 38 days with fluorouracil on days 1–4 and 29–32 by continuous intravenous infusion and either mitomycin as bolus on day 1 only or cisplatin by infusion on days 1 and 29.^7^ Patients randomly allocated to receive maintenance were given two additional courses of fluorouracil and cisplatin on days 71–74 and 92–95 after the start of chemoradiotherapy—ie, weeks 11 and 14. Before treatment, patients were staged according to the UICC 1990 staging system.^12^ Abdominopelvic CT scans and chest radiographs or thoracic CT scans were mandated, but not MRI or PET.

There were three primary tumour assessments (figure 1), made by the patient's clinician (single clinical oncologist review). Assessment 1 (11 weeks from the start of chemoradiotherapy) was timed to allow any adverse events from radiotherapy to resolve and before patients randomly assigned to maintenance treatment started therapy. Assessment 2 was at 18 weeks from the start of chemoradiotherapy (4 weeks after completion of maintenance therapy for those receiving it) to assess the effect of maintenance therapy. Assessment 3 was at 26 weeks from the start of chemoradiotherapy in case of treatment delay and this timepoint has been used in other squamous cell cancers to allow for tumours that relapse or progress early.^13^ Information about examination under anaesthetic was not collected during the trial and biopsies were not routinely done unless there was a high suspicion of residual disease because of anxieties from the radiation oncologist regarding healing in an irradiated area (according to UK practice). Patients who did not have a complete clinical response (those with partial response, stable disease, or progressive disease who did not have salvage surgery before week 26) at either assessment 1 or 2 could have subsequent assessments and delay interventions at the time (to determine slow responders). Patients diagnosed with progressive disease at assessment 1 or any other time before 26 weeks could still have assessment 2 or 3, but as their complete clinical response status could be influenced by any salvage treatment received they were excluded from all analyses.

Response was assessed according to Response Evaluation Criteria in Solid Tumors (version 1.0).^14^ Digital rectal examination was done at all three assessments, with mandatory abdominopelvic CT scan and chest radiograph or whole body CT scan at assessment 3 (figure 1). Residual or recurrent disease was confirmed by biopsy before further therapy if results from other evaluations were ambiguous.

Patients were classified into two groups at each assessment: patients with a complete clinical response or patient without a complete clinical response (ie, patients with a partial response, stable disease, or disease progression). Patients who attended the assessments with insufficient response data were classified as “unknown” whereas those who either did not attend assessments or whose data were not reported were classified as “missing”. Where possible, the missing nodal status was extrapolated from the most recent previous and subsequent assessments or follow-up information. In contrast to our previous publication of ACT II trial data,^7^ which defined complete response according to primary disease status only, in this report we included absence of nodal disease in the definition of complete clinical response to more accurately describe a group of patients with complete disappearance of tumour.

### Outcomes

In the main trial evaluating chemoradiotherapy the primary endpoints were complete clinical response at 26 weeks from the start of chemoradiotherapy, acute toxic effects for patients that received chemoradiotherapy and progression-free survival for patients that received maintenance chemotherapy. This post-hoc analysis investigated complete clinical response at all three assessments (11 weeks, 18 weeks, and 26 weeks from the start of chemoradiotherapy) as well as progression-free survival and overall survival measured from the time of randomisation.

### Statistical analysis

The association between tumour response and progression-free survival or overall survival at each timepoint was examined by Kaplan-Meier curves and Cox regression models. Crude and baseline adjusted hazard ratios (HRs) for achieving versus not achieving complete clinical response were calculated and adjusted for prognostic baseline factors and trial treatment. Progression-free survival events were defined as progressive disease, local recurrence (with or without metastases), metastases, or death from any cause. New tumours were not defined as progression-free survival events. Overall survival events included deaths from any cause. Time-to-event endpoints were measured from the date of randomisation, and patients without the event of interest were censored at date of last follow-up. Sensitivity analyses were done to check the effect of extrapolating nodal status when missing on the proportion of patients with a complete response.

To ensure that the analyses were not biased by the inclusion of patients who died before assessment 3, progression-free survival and overall survival were analysed both in all randomised patients using complete clinical response status where known, and those patients who attended clinic at all three timepoints and did not have salvage treatment before assessment 3.

Two sensitivity analyses of progression-free survival and overall survival was done using two extreme assumptions. The first was done on all randomised patients, and where response status was unknown it was assumed to be complete clinical response, and the second was done on all randomised patients, and where response status was unknown it was assumed to be not- complete clinical response. Analyses other than overall survival and progression-free survival were based on those patients who had tumour assessment data at all timepoints excluding those patients who had salvage treatment, to have a uniform dataset for these analyses, unaffected by missing tumour response data. All reported p values are two-sided, and analyses were done using Stata (version 12). We also examined the prognostic performance of complete clinical response status at each timepoint, using sensitivity and false-positive rates. This study is registered as at ISRCTN, number 26715889.

### Role of the funding source

The funder had no role in study design, report writing, or collecting, analysing, or interpretation of the data. RG-J, DS-M, HMM, RB, SB, AH, and LK had full access to the data. All authors made the decision to submit the report for publication and gave final approval for submission.

## Results

940 patients were enrolled from June 4, 2001, until Dec 16, 2008. The baseline characteristics of all individuals enrolled in the trial have been reported previously.^7^ Overall, the median age was 58 years, 486 (52%) of 940 had a primary tumour of 5 cm or smaller (T1 or T2), 430 (46%) of 940 had a primary tumour larger than 5 cm or invasion to neighbouring organs (T3 or T4), 305 (32%) of 940 had positive lymph nodes, 787 (84%) of 940 had a tumour in the anal canal, and 132 (14%) of 940 had a tumour in the anal margin. Of the 940 patients, 249 were excluded for subgroup analysis: 241 patients did not attend all tumour assessments, or had results where it was not possible to determine response status, and eight patients had salvage treatment before the third assessment. The baseline characteristics of these 691 remaining patients who were analysed in this subgroup analysis were similar to both the entire trial population 940 patients (appendix p 1), and the 249 patients who were excluded (appendix p 2). The proportions of patients with primary tumour response data at all three assessments were similar in the two treatment groups (345 [73%] of 472 patients who received mitomycin *vs* 346 (74%) of 468 patients who received cisplatin; appendix p 3). Eight of 19 patients with confirmed early progressive disease at assessments 1 or 2 had a potentially curative resection before week 26 and therefore had a complete clinical response at the third assessment (week 26). Excluding these patients in the analysis had a negligible effect on the results.

The median follow-up, censoring deaths, was 5·1 years (IQR 3·9–6·9) for all 940 patients and 5·2 years (4·0–6·8) for the 691 patients who attended all three assessments. There were 211 deaths and 292 progression-free survival events in the whole trial population. 23 of these deaths occurred before assessment 3. 12 patients died before assessment 1 (six from chemotherapy-related adverse events, two from anal cancer, three from reasons unrelated to cancer, and one from an unknown cause). Three died between assessments 1 and 2 (two by reasons unrelated to cancer and one from an unknown cause). Eight patients died between assessments 2 and 3 (six from anal cancer, one from radiotherapy, and one by suicide). There were 127 deaths and 182 progression-free survival events in the subgroup of 691 patients who had attended all three assessments.

Compliance to chemoradiotherapy in the subgroup pf 691 patients who were assessable at all three timepoints was high and similar between the mitomycin and cisplatin groups (appendix p 4) and similar to that for all 940 trial patients.^7^ The median overall treatment time was 38 days (IQR 38–39 days) in both the mitomycin and cisplatin groups.

In our subgroup analysis of the 691 patients with response data at all three timepoints, the proportion of patients with complete clinical response increased over time: 441 (64%, 95% CI 61–67) of 691 patients had a complete clinical response at assessment 1, 556 (81%, 78–88) at assessment 2, and 590 (85%, 83–88) at assessment 3 (Table 1, Table 2). 421 (95%) of 441 patients who had a complete clinical response at assessment 1 maintained this complete clinical response at assessment 2 and 411 (93%) of 441 still had a complete clinical response at assessment 3 (table 3). The remaining 30 (7%) of 441 patients either had a suspected relapse at assessment 2 or 3 (n=25) or they were missing data for nodal status (n=5). Complete clinical response was achieved in 492 (52%) of 940 patients at assessment 1 (11 weeks), 665 (71%) of patients at assessment 2 (18 weeks), and 730 (78%) of patients at assessment 3 (26 weeks). However, 151 (72%) of 209 patients who were not in complete clinical response at assessment 1 achieved complete clinical response by assessment 3, and 115 (76%) of 151 were alive and disease-free on last follow-up after treatment. Therefore, 115 (55%) of 209 patients who did not have a complete clinical response at assessment 1 could be considered slow responders.

Of the overall trial population, 119 (13%) of 940 patients did not have a complete clinical response at assessment 3 (table 3). Of these 119, two (2%) patients had defunctioning stomas for side-effects of radiotherapy, 27 (23%) had salvage surgery (abdominoperineal excision of rectum or anorectal excision), and three had other types of surgery (all done after 26 weeks). Disease was pathologically confirmed before radical surgery.

The difference in the population of patients achieving a complete clinical response between patients in the cisplatin and mitomycin groups or between patients in the groups that received or did not receive maintenance was not significant at any assessment (table 1). There was no interaction between maintenance treatment and mitomycin or cisplatin (p=0·88 for both). The proportion of patients with a complete clinical response at assessment 3 (disregarding nodal status), was 311 (90%) of 345 for mitomycin and 313 (91%) of 346 for cisplatin (appendix p 3), similar to those among all 940 patients as previously reported (391 [91%] of 432 patients treated with mitomycin and 386 [90%] of 431 patients treated with cisplatin).^7^ When nodal status was included at assessment 3 (26 weeks), the results were similar, with 290 (84%) of 345 patients in the mitomycin group achieving a complete clinical response compared with 294 (85%) of 346 patients in the cisplatin group (appendix p 3).

**Figure 2:**
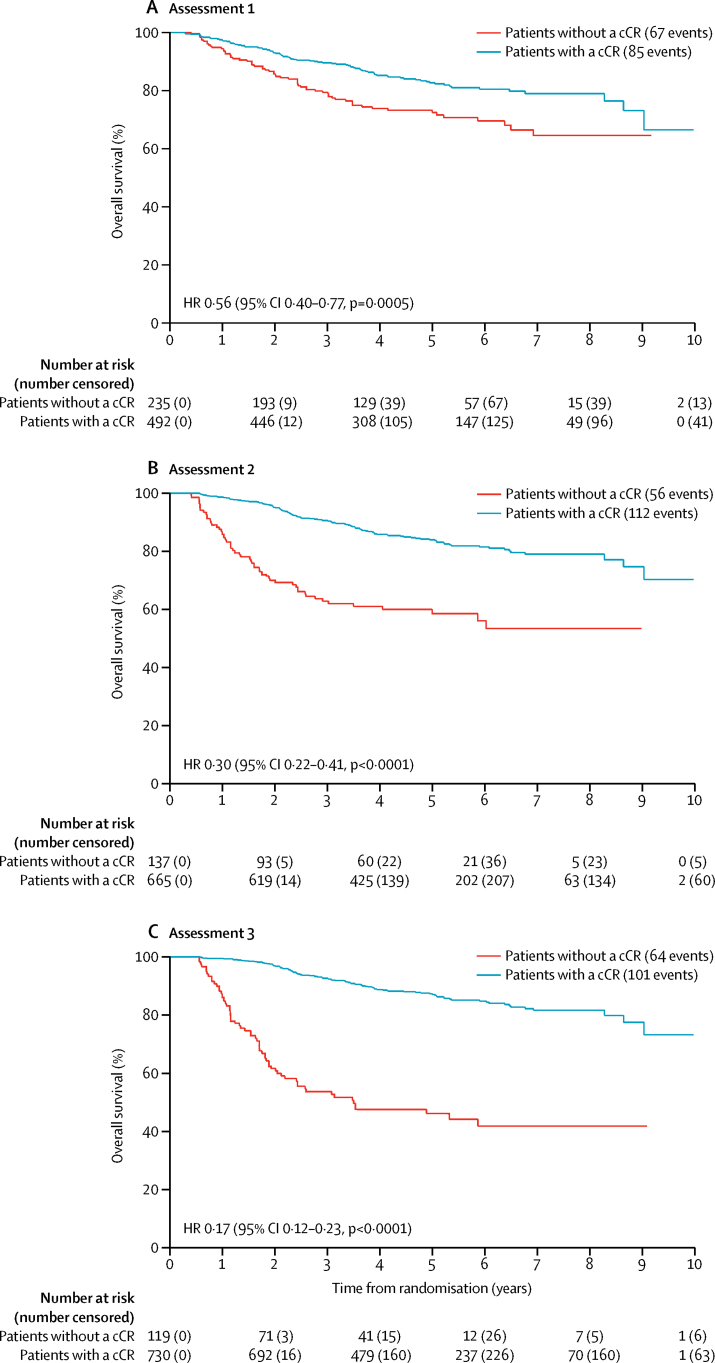
Overall survival according to response at assessments 1, 2, and 3 among all 940 patients in whom response data were known cCR=complete clinical response. HR=hazard ratio.

Regardless of when patients were assessed, clinical complete response was affected by patient's tumour size and nodal stage (appendix p 5). Although the clinical complete response was significantly affected by tumour metastasis to neighbouring organs (compared with no tumour metastasis) at assessment 1 (p=0·0009), there was no longer a significant difference between patients with and without metastasis to neighbouring organs at assessment 3 (p=0·08; appendix p 5). Clinical complete response was unaffected by age (patients aged 65 years and older compared with patients younger than 65 years). However, clinical complete response was affected by a patient's sex at assessment 3, but not at assessment 1 (appendix p 5). Overall survival of the overall trial population was analysed (with tumour response data, where available at any of the three assessments; figure 2). Table 4 shows the crude and adjusted HRs for overall survival. The 5 year overall survival for patients with complete clinical response and patients without a complete clinical response groups were: 83% (79–86) and 72% (66–78) at assessment 1; 84% (81–87) and 59% (49–67) at assessment 2; and 87% (84–89) and 46% (37–55) at assessment 3. The overall survival for the subgroup of 691 patients who had tumour assessments at all three timepoints was also assessed (appendix p 8) and was similar to that found in the overall trial population (table 4). The 5 year overall survival in this subgroup for patients with a complete clinical response and patients without a complete clinical response group was 85% (95% CI 81–88) and 75% (68–80) at assessment 1, 86% (82–88) and 61% (50–70) at assessment 2, and 87% (84–90) and 48% (36–58) at assessment 3.

**Figure 3:**
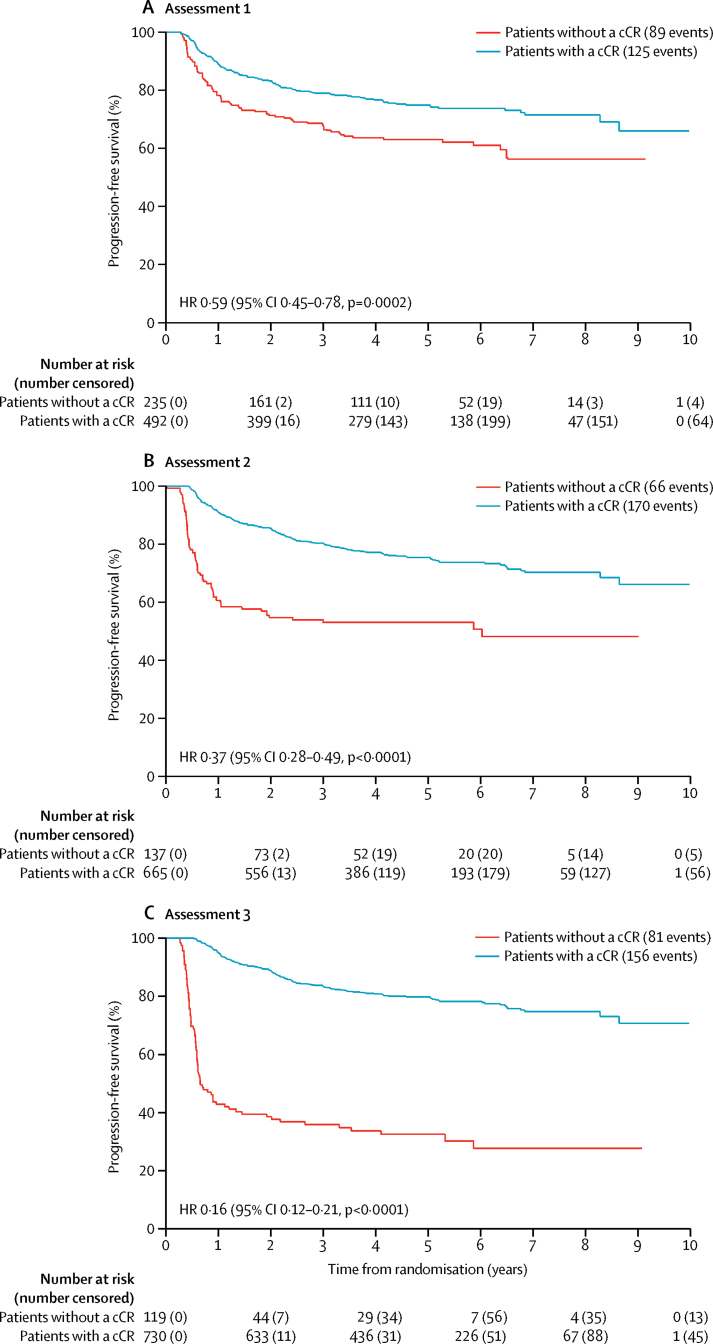
Progression-free survival according to response at assessments 1, 2, and 3 among all 940 patients in whom response data were known cCR=complete clinical response. HR=hazard ratio.

Progression-free survival for the overall trial population and the subgroup of patients with a response at all timepoints was also analysed (table 4, figure 3, appendix p 9). The 5 year progression-free survival in patients who had a complete clinical response compared with those who did not have a complete clinical response was 75% versus 63% at assessments 1, 75% versus 53% at assessment 2, and 80% versus 33% at assessment 3. Analysis of the subgroup of 691 patients who had data for all three timepoints yielded similar results (appendix p 9). There was no difference in progression-free survival events in patients who had a complete clinical response at assessment 1 (105 events in 441 patients) and those who had a complete clinical response only at assessment 3 (nine events in 43 patients) (absolute difference 2·9 [95% CI −9·9 to 15·7], p=0·67).

We did several analyses for overall survival and progression-free survival to check for consistency in the results because 241 of 940 patients had missing tumour assessments at one or more timepoints (where the complete clinical response status was unavailable: unknown or missing) and eight patients who had available data were excluded because they had salvage treatment. Sensitivity analyses on the basis of imputing data for missing tumour response (with assumptions) and those who did not attend assessments (which includes those for which response data were not reported) provided similar results for both overall survival and progression-free survival as the main analysis (table 4, appendix pp 10–13); as well as for imputation for missing nodal status, assumed to be either positive or negative (appendix p 6). Furthermore, after excluding 23 deaths occurring before assessment 3 (which would otherwise bias the group of patients with missing data), there was no difference in overall survival between patients who attended, patients who did not attend this assessment, or those with data not reported (table 3). At 1 year the overall survival was 96% (95% 95–98) for 809 patients who attended assessment 1 versus 91% (84–95) for patients who either did not attend or had data unreported at assessment 1. 1 year overall survival at assessment 2 was 97% (95–98) for the 852 patients who attended compared with 90% (80–95) for 73 patients who did not attend or had data not reported, and 1 year overall survival at assessment 3 was 98% (96–99) for the 871 patients who attended versus 84% (70–92) for the 46 patients who did not attend or data not reported at assessment 3 (data not shown).

The sensitivity (ie the number of patients with a complete response who are alive relative to the total number of patient alive) of complete clinical response to predict overall survival was analysed at all three timepoints (appendix p 7). Sensitivity of complete clinical response to predict overall survival at 1 year was the highest at assessment 3 (88%) compared with at assessment 2 (85%) or assessment 1 (68%). The probability of a false-positive at 1 year was also the lowest at assessment 3 (20%) compared with assessment 2 (32%) and assessment 1 (50%).

## Discussion

Our results show that the proportion of those with a complete clinical response at 26 weeks (assessment 3) is more informative than either of the two earlier assessments, and that it is acceptable to monitor partial responders carefully up to the 26 week assessment. This extended period with careful monitoring has not been international practice to date, and there are patients who have salvage surgery because of residual tumour found shortly after completing chemoradiotherapy. Therefore it is important for patients and clinicians to establish the best time to assess tumour response, and we have done so with data from our clinical trial.

There was no evidence that maintenance therapy acted as a confounding factor for the association between complete clinical response status and overall survival and progression-free survival, as maintenance therapy did not influence whether a patient had complete clinical response. Moreover, maintenance treatment started only after assessment 1, there was no difference in the complete clinical response rates between patients with and without maintenance therapy (table 1), and there was no effect of maintenance therapy on either progression-free survival or overall survival, as detailed in our original report on this trial.^7^ Since the median overall treatment time was the same in both the mitomycin and cisplatin groups (38 days [IQR 38–39 days]) the timing of assessment of complete clinical response was probably not confounded by variations in treatment duration. There was a substantial increase in the proportion of patients with a complete clinical response at 26 weeks (assessment 3) from the start of chemoradiotherapy compared with earlier assessments (table 1). These data are compatible with the RTOG-8704 trial results,^1^ which mandated a biopsy after chemoradiotherapy at 4–6 weeks after completion of chemotherapy as part of the assessment and showed that the combination of mitomycin and fluorouracil with radiation produced a pathological complete response in 92% of patients at 6 weeks after completing chemoradiotherapy.^1^ Patients with residual cancer shown in the biopsy after chemoradiotherapy were treated with a salvage regimen of additional pelvic radiotherapy (9 Gy), fluorouracil, and cisplatin (100 mg/m^2^). Of the 24 assessable patients who had salvage chemoradiotherapy, 12 (50%) were rendered disease-free. Our results imply that simply waiting longer might have achieved similar results. Our findings are also consistent with those previously described in a series of sequential chemoradiotherapy studies with mitomycin,^15^ which showed that some patients with squamous cell carcinoma of the anus required 9–12 months to achieve a complete clinical response (defined as complete resolution of all clinical signs of the primary cancer for a duration of 2 months; if not, the tumour was scored as residual).

A limitation of our analysis is that the group which achieved a complete clinical response at assessment 3 (week 26) does not truly represent all responders to chemoradiotherapy because it also includes patients who have had an early and sustained response and those who have a late response, but not patients who had a complete clinical response at assessment 1 but subsequently relapsed or did not have a complete clinical response by assessment 3.

Eventual outcomes (overall survival and progression-free survival) were independent of the timing that complete clinical response was achieved. These outcomes seem to be independent of whether complete clinical response was achieved at assessment 3 or at assessment 1. The HRs from both the overall survival and progression-free survival curves suggest that assessment 3 (26 weeks from the start of treatment) gives the most widely discriminating effect on survival outcomes, and suggests this is the optimum timepoint for assessment. Tumour assessment (including nodal status) at week 26 should therefore be explored as a surrogate endpoint for progression-free survival and overall survival in future trials.

These findings challenge guidelines^8^, ^9^ for the post-treatment follow-up of patients with anal cancer, which include serial digital rectal examination, with biopsy of clinically suspicious lesions recommended at weeks 4–6, 6–8, and 8–12 after chemoradiotherapy completion, respectively. In our trial, not all 940 randomised patients had a known complete clinical response status at all three assessment timepoints. To address whether substantial bias could have arisen we made extreme assumptions about unknown status in two sensitivity analyses, and both produced the same conclusions irrespective of whether we considered the whole trial population or only those who attended all three assessments—ie, that the strongest association between complete clinical response status and either overall survival or progression-free survival is at assessment 3.

Anal cancers can regress slowly after completion of chemoradiotherapy treatment. Accurate identification of response is a crucial component to optimise patient management. Clinical groups in the USA have recommended salvage surgery should only be considered after at least 12 weeks because complete resolution might take 3–6 months.^1^ The ideal method and timing of response evaluation and when maximum response occurs after chemoradiotherapy is unclear. Retrospective reports suggested initial early clinical response is an independent prognostic factor for survival.^16^, ^17^, ^18^ Randomised studies have done clinical assessments of the tumour between 6 weeks and 12 weeks after completion of chemoradiotherapy.^1^, ^2^, ^3^, ^4^, ^5^, ^6^

The mainstay of clinical evaluation has relied on digital rectal examination and careful examination of the inguinal regions. The limitations of this approach include the subjectivity of the clinical examination and the absence of treatment response information for any more deeply sited, non-palpable disease, including the pelvic lymph nodes. Also, radiotherapy-induced acute local effects can cause problems in our interpretation of response. Severe skin reactions, oedema, residual fibrosis, or scar tissue can be difficult to distinguish from persistent, active, or recurrent disease.^19^, ^20^ The risk of radionecrosis should also be kept in mind when considering biopsy. A significant correlation has also been observed between high-grade acute toxicity in terms of skin reactions, cystitis, proctitis, or enteritis (National Cancer Institute Common Toxicity Criteria grade 3 or worse) during chemoradiotherapy and overall survival, locoregional control, and stoma-free survival.^21^

Imaging in terms of endoanal ultrasound or MRI has an established role in initial locoregional staging of the primary and pelvic lymph nodes,^8^, ^10^ but their ability to assess response accurately is less welldefined.^22^ It can take 16–20 weeks to allow sufficient time for resolution of oedema to enable accurate classification of treatment response by ultrasound.^23^ MRI complements clinical assessment but can assign a worse stage prognosis to a patient, particularly lymph node status because enlarged lymph nodes can be recorded as node positive although many do not contain tumours. Metabolic and functional imaging techniques, such as diffusion-weighted MRI,^24^ or PET with ^18^F-fluorodeoxyglucose,^25^, ^26^, ^27^ might offer an alternative option to assess response at an early timepoint without the potential morbidity of a biopsy, but large prospective studies are required.

The reported 5 year overall survival with salvage abdominoperineal resection for persistent or locally recurrent anal cancer was only 24–64%;^28^, ^29^ hence, the rationale for proactive follow-up. For surgeons, there is always a tension between doing an early biopsy to define an early recurrence with limited locoregional disease, when surgical salvage is likely to be effective, and the risk of provoking necrosis from a biopsy after chemoradiotherapy. 209 (30%) of 691 patients were not in complete clinical response 4 weeks after chemoradiotherapy (assessment 1); however, 115 remained disease-free at later follow-up, hence, early surgical salvage would not have been appropriate for these patients.

The RTOG-8704 trial mandated biopsy of the primary tumour at 4–6 weeks after a dose of 45 Gy to confirm complete pathological response in clinically residual disease,^1^ but routine biopsy is controversial in the monitoring of response to treatment, with some clinicians supporting random biopsies at a 3 month interval, while others support a biopsy only when there is a suspicious lesion.^30^

Our results suggest that early response assessments might not be reliable by failing to account for patients who are slow to respond to treatment. The data confirm that partial regression can be managed by close follow-up to confirm eventual complete regression. Others have recommended that patients deemed to be slow to respond (persistence of local disease with regression <50%) should be evaluated by at least two physicians at the end of radiotherapy, patients should be given an additional one or two courses of chemotherapy courses.^31^ However, our data previously showed no significant effect from an additional two cycles of maintenance chemotherapy (fluorouracil and cisplatin) after chemoradiotherapy (complete clinical response 82% *vs* 85% p=0·34).^7^

The ACT II chemoradiotherapy schedule achieved an excellent proportion of patients with an early complete clinical response, but our results show 151 (72%) of 209 patients not in complete clinical response at 4 weeks after chemoradiotherapy achieved complete clinical response at 26 weeks. The consistency of the overall treatment time (median 38 days) makes the validity of our data for timing of complete clinical response assessment even stronger.

Although we would advise careful monitoring from completion of treatment to facilitate timely surgical salvage therapy for progressive disease, it seems safe to observe a resolving tumour up to 26 weeks after the start of chemoradiotherapy, and some patients could thus avoid unnecessary surgery. It might even be safe to extend evaluation beyond this timepoint, as some studies suggest a few patients might require more than 10 months for complete regression, but prospective data are required to confirm this timeline. We propose guidelines should be revised, and that the assessment of response at 26 weeks be used in future trials and explored as a surrogate endpoint for overall survival and progression-free survival.

## Figures and Tables

**Table 1:** Complete clinical response at all three assessments in patients with primary tumour response data at all three assessments

	**Overall (n=691)**	**Mitomycin (n=345)**	**Cisplatin (n=346)**	χ^2^	No maintenance (n=347)*	**Maintenance (n=305)**	χ^2^
Assessment 1	441 (64%); (61–67)	231 (67%); (62–72)	210 (61%); (56–66)	p=0·09	224 (65%); (60–70)	187 (61%); (56–67)	p=0·39†
Assessment 2	556 (80%); (78–88)	273 (79%); (75–83)	283 (82%); (78–86)	p=0·38	274 (79%); (75–83)	252 (83%); (78–87)	p=0·24
Assessment 3	590 (85%); (83–88)	292 (85%); (81–88)	298 (86%); (82–90)	p=0·58	294 (85%); (81–89)	264 (87%); (83–90)	p=0·51

Data are shown as n (%); (95% CI), excluding eight patients who had salvage surgery.

* 39 of 691 patients did not get randomly assigned to the maintenance therapy (at physicians' discretion), so they were not included in the maintenance analysis. The total number analysed for maintenance comparison is, therefore, 652 and not 691.

† No difference in the proportion of patients with a complete response is expected between patients with and without maintenance therapy at assessment 1 because maintenance treatment would only start after this time. The p values shown were calculated with χ^2^ tests.

**Table 2:** Distribution of patients and tumour response for patients who attended all three assessments (n=691)

	**Patients with complete clinical response**	**Patients without complete clinical response**	Patients with unknown response data*
Assessment 1	441	209	41
Assessment 2	556	106	29
Assessment 3†	590	88	13

* Patients classified as “unknown” attended the assessment but had response data that were inconclusive.

† 23 patients died before assessment 3. Some patients did not attend for more than one assessment or had missing response data for more than one assessment so it is not possible to sum these numbers over all three timepoints.

**Table 3:** Distribution of patients and tumour response for all patients in the trial (n=940)

	**Patients with complete clinical response**	**Patients without complete clinical response**	Patients with unknown response data*	**Patients with missing data**†
Assessment 1	492	235	82	131
Assessment 2	665	137	50	88
Assessment 3‡	730	119	22	69

* Patients classified as “unknown” attended the assessment but had response data that were inconclusive.

† Patients classified as “missing” included those for whom response data were not reported and patients who did not attend clinic for assessment.

‡ 23 patients died before assessment 3. Some patients did not attend for more than one assessment or had missing response data for more than one assessment so it is not possible to sum these numbers over all three timepoints.

**Table 4:** Association between overall survival or progression-free survival and tumour response at three different assessment timepoints

	**Overall survival**		**Progression-free survival**	
	Crude	Adjusted*	Crude	Adjusted*
**All 940 patients (using response data wherever available)**				
1	0·56 (0·40–0·77); p<0·0005	0·77 (0·50–1·18); p=0·22	0·59 (0·45–0·78); p<0·002	0·66 (0·46–0·95); p=0·02
2	0·30 (0·22–0·41); p<0·0001	0·40 (0·26–0·61); p<0·0001	0·37 (0·28–0·49); p<0·0001	0·43 (0·29–0·62); p<0·0001
3	0·17 (0·12–0·23); p<0·0001	0·22 (0·14–0·35); p<0·0001	0·16 (0·12–0·21); p<0·0001	0·15 (0·10–0·21); p<0·0001
**691 patients with response data at all three timepoints**†				
1	0·55 (0·39–0·79); p=0·001	0·81 (0·51–1·29); p=0·38	0·61 (0·45–0·82); p=0·001	0·68 (0·46–0·99); p=0·05
2	0·30 (0·20–0·43); p<0·0001	0·44 (0·26–0·72); p=0·001	0·36 (0·26–0·50); p<0·0001	0·44 (0·29–0·66); p<0·0001
3	0·17 (0·12–0·24); p<0·0001	0·24 (0·14–0·40); p<0·0001	0·15 (0·11–0·21); p<0·0001	0·16 (0·10–0·24); p<0·0001
**All 940 patients (missing response data assumed to be complete clinical response)**				
1	0·72 (0·54–0·96); p=0·028	0·98 (0·66–1·46); p=0·93	0·71 (0·55–0·92); p=0·01	0·79 (0·56–1·11); p=0·17
2	0·38 (0·28–0·52); p<0·0001	0·46 (0·30–0·70); p<0·0001	0·44 (0·33–0·58); p<0·001	0·46 (0·32–0·66); p<0·0001
3	0·25 (0·19–0·34); p<0·0001	0·31 (0·20–0·47); p<0·0001	0·21 (0·16–0·27); p<0·001	0·19 (0·13–0·27); p<0·0001
**All 940 patients (missing response data assumed to be not complete clinical response)**				
1	0·51 (0·39–0·68); p<0·0001	0·68 (0·47–0·97); p=0·04	0·57 (0·45,0·72); p<0·0001	0·65 (0·48–0·89); p=0·01
2	0·31 (0·24–0·41); p<0·0001	0·40 (0·28–0·59); p<0·0001	0·39 (0·31,0·49); p<0·0001	0·49 (0·35–0·68); p<0·0001
3	0·16 (0·12–0·20); p<0·0001	0·19 (0·13–0·27); p<0·0001	0·16 (0·13–0·21); p<0·0001	0·17 (0·12–0·24); p<0·0001

Data are HR (95% cCI); p value. Complete clinical response is the complete disappearance of disease in the primary and nodes.

* Adjusted for potential confounding factors: age, sex, site of primary, tumour differentiation, histology, baseline white blood cell count, baseline platelets, baseline haemoglobin, tumour size, nodal stage, and trial treatment.

† Excluding eight patients who had salvage surgery.
