# Correction to *Lancet Oncol* 2017; 18: 347–56

**DOI:** 10.1016/S1470-2045(17)30173-0

**Published:** 2017-04

*Glynne-Jones R, Sebag-Montefiore D, Meadows HM, et al. Best time to assess complete clinical response after chemoradiotherapy in squamous cell carcinoma of the anus (ACT II): a post-hoc analysis of randomised controlled phase 3 trial.* Lancet Oncol *2017; **18**: 347–56*—This Article should have been published under the copyright “© The Authors. Published by Elsevier Ltd. This is an Open Access article under the CC BY license”. This correction has been made to the online version as of March 2, 2017.

