# Supplementary material for: Best time to assess complete clinical response after chemoradiotherapy in squamous cell carcinoma of the anus (ACT II): a post-hoc analysis of randomised controlled phase 3 trial
Source: Lancet Oncol. 2017 Mar;18(3):347–56. doi: 10.1016/S1470-2045(17)30071-2 (PMC5337624; doi:10.1016/S1470-2045(17)30071-2)
Supplement: Supplementary appendix [file mmc1.pdf]

THE LANCET
Oncology

Supplementary appendix

This appendix formed part of the original submission and has been peer reviewed. We post it as supplied by the authors.

Supplement to: Glynne-Jones R, Sebag-Montefiore D, Meadows HM, et al, on behalf of the ACT II study group. Best time to assess complete clinical response after chemoradiotherapy in squamous cell carcinoma of the anus (ACT II): a post-hoc analysis of randomised controlled phase 3 trial. *Lancet Oncol* 2017; published online Feb 10. http://dx.doi.org/10.1016/S1470-2045(17)30071-2.

Appendix

Table 1A. Baseline characteristics of 691 patients who attended clinic for tumour assessment at all 3 time-points.

**Patient characteristic,** N=total randomised to each arm	**MMC No maintenance N=199**	**CisP No maintenance N=187**	**MMC Maintenance N=146**	**CisP Maintenance N=159**
**Age years, median**	60.2	57.4	56.9	58.3
(IQR)	(52.5 to 65.2)	(50.7 to 65.3)	(49.9 to 65.2)	(51.2 to 67.1)
<65	147 (74)	140 (75)	109 (75)	111 (70)
≥65	52 (26)	47 (25)	37 (25)	48 (30)
**Gender**				
Female	126 (63)	121 (65)	89 (61)	104 (65)
Male	73 (37)	66 (35)	57 (39)	55 (35)
**Site of primary**				
Canal	164 (82)	160 (86)	115 (79)	138 (87)
Margin	30 (15)	26 (14)	28 (19)	17 (11)
Not reported	5 (3)	1 (<1)	3 (3)	4 (3)
**T stage**				
T1	18 (9)	19 (10)	13 (9)	16 (10)
T2	85 (43)	86 (46)	56 (38)	75 (47)
T3	66 (33)	53 (28)	52 (36)	47 (30)
T4	23 (12)	27 (14)	20 (14)	20 (13)
TX	7 (4)	2 (1)	5 (3)	1 (<1)
T1+T2	103 (52)	105 (56)	69 (47)	91 (57)
T3+T4	89 (45)	80 (43)	72 (49)	67 (42)
**Nodal status**				
Negative	129 (65)	122 (65)	88 (60)	105 (66)
Positive	61 (31)	59 (32)	53 (36)	49 (31)
NX	9 (5)	5 (3)	5 (3)	5 (3)
Not reported	0	1 (<1)	0	0
**GFR**				
<60	8 (4)	10 (5)	5 (3)	5 (3)
≥60	191 (96)	177 (95)	141 (97)	154 (97)
**Differentiation**				
Well	26 (13)	32 (17)	19 (13)	20 (13)
Moderate	77 (39)	77 (41)	66 (45)	65 (41)
Poor	61 (31)	54 (29)	42 (29)	50 (31)
Unknown	35 (18)	24 (13)	19 (13)	24 (15)
**Tumour type**				
Basaloid	24 (12)	21 (11)	18 (12)	19 (12)
Cloacogenic	4 (2)	2 (1)	1 (<1)	1 (<1)
Squamous	162 (81)	155 (83)	119 (82)	132 (83)
Unknown	9 (5)	9 (5)	8 (5)	7 (4)
**Pre-treatment colostomy**				
No	178 (89)	159 (85)	125 (86)	141 (89)
Yes	20 (10)	28 (15)	21 (14)	17 (11)
Not reported	1 (<1)	0	0	1 (<1)

Overall rates: median age: 58 years, T1+T2: 53%, T3+T4: 45%, positive lymph nodes: 32%, Tumour in the anal canal: 84% and in the margin: 15%, median follow-up censoring deaths: 5.2 years.

Table 1B. Baseline characteristics of 249 patients whose response status could not be classified as cCR or non-cCR, who did not attend clinic for tumour assessment, or response data was not reported, at all 3 time-points or who had salvage surgery before week 26

**Patient characteristics**	**MMC No maintenance** N=47	**CisP No maintenance** N=59	**MMC Maintenance*** N=80	**CisP Maintenance*** N=63
		N (%)		
**Age**				
years median (IQR)	57.2 (49.8 to 64.5)	56.7 (50.3 to 66.4)	58.2 (50.3 to 66.4)	55.2 (46.6 to 63.5)
<65	37 (79)	44 (75)	57 (71)	54 (86)
≥65	10 (21)	15 (25)	23 (29)	9 (14)
**Gender**				
Female	27 (57)	32 (54)	52 (65)	36 (57)
Male	20 (43)	27 (46)	28 (35)	27 (43)
**Site of primary**				
Canal	38 (81)	48 (81)	72 (90)	52 (83)
Margin	8 (17)	7 (12)	7 (9)	9 (14)
Not reported	1 (2)	4 (7)	1 (1)	2 (3)
**T stage**				
T1	7 (15)	5 (8)	8 (10)	5 (8)
T2	14 (30)	23 (39)	31 (39)	25 (40)
T3	14 (30)	18 (31)	27 (34)	18 (29)
T4	10 (21)	7 (12)	13 (16)	15 (24)
TX	2 (4)	6 (10)	1 (1)	0
T1+T2	21 (45)	28 (47)	39 (49)	30 (48)
T3+T4	24 (51)	25 (42)	40 (50)	33 (52)
**Nodal status**				
Negative	27 (57)	33 (56)	53 (66)	30 (48)
Positive	16 (34)	19 (32)	20 (25)	28 (44)
NX	9 (5)	5 (3)	5 (3)	5 (3)
Not reported	4 (9)	7 (12)	7 (9)	5 (8)
**GFR**				
<60	5 (11)	2 (3)	7 (9)	3 (4)
≥60	42 (89)	57 (97)	73 (91)	60 (95)
**Differentiation**				
Well	3 (6)	6 (10)	6 (8)	9 (14)
Moderate	23 (49)	25 (42)	38 (48)	24 (38)
Poor	14 (30)	19 (32)	18 (23)	19 (30)
Unknown	7 (15)	9 (15)	18 (23)	11 (17)
**Tumour type**				
Basaloid	4 (9)	4 (7)	11 (14)	7 (11)
Cloacogenic	1 (2)	3 (5)	2 (3)	0
Squamous	39 (83)	46 (78)	63 (79)	53 (84)
Unknown	3 (6)	6 (10)	4 (5)	3 (5)
**Pre-treatment colostomy**				
No	35 (74)	51 (86)	66 (83)	51 (81)
Yes	11 (23)	8 (14)	14 (18)	12 (19)
Not reported	1 (2)	0	0	0

*3 in the MMC and 4 in the CisP arms were not randomised to maintenance or no maintenance arms

Table 2. Response at 26 weeks from the start of CRT (Assessment 3) among 691 patients with tumour assessments at all 3 time-points.

	MMC	CisP	No Maintenance	Maintenance
		N (%)		
**Patients with primary tumour response data at all three assessments (N=691)**	**345*/472 (73%)**	**346*/468 (74%)**	**347*/446 (78%)**	**305*/448 (68%)**
CR[¶]				
CR N0 (cCR)	290 (84.1)	294 (85.0)	290 (83.6)	262 (85.9)
CR N+	12 (3.5)	9 (2.6)	11 (3.2)	6 (2.0)
CR Nx	9 (2.6)	10 (2.9)	12 (3.5)	6 (2.0)
PR	11 (3.2)	15 (4.3)	13 (3.8)	13 (4.3)
SD	4 (1.2)	5 (1.4)	5 (1.4)	4 (1.3)
PD	19 (5.5)	13 (3.8)	16 (4.6)	14 (4.6)
Nx extrapolated[#]	2/9	4/10	4/12	2/6
CR N0 (cCR) including extrapolated Nx	292 (84.6)	298 (86.1)	294 (84.7)	264 (86.6)

CR: complete response in the primary only; cCR: CR and node negative (N0); N+: node positive; Nx: nodal status missing; PR: partial response; SD: stable disease; PD: progressive disease.

*Percentages based on these numbers

[¶]The absolute difference in CR N0 between CisP and MMC arms is 0.9% (95% CI: -4.5 to +6.3, P-value=0.74) and in CR (response in primary alone, nodes ignored) is 0.3% (-4.1 to +4.7, P-value=0.89) and for CR N0 between Maintenance and No Maintenance arms is 2.3% (95% CI: -3.2 to +7.8, p-value=0.41) and for CR is 0.4% (-5.0 to , +4.3, P-value=0.88).

[#] Nodal status missing for 56, 59 and 19 patients at Assessments 1, 2 and 3 respectively. Missing nodal status data for these patients were extrapolated from the known nodal status at previous and subsequent assessments wherever possible. 46 patients were not randomised in the maintenance comparison.

Table 3. Compliance to treatment among 691 patients who had tumour assessment data at all 3 time-points

	**MMC** (N=345)	**CisP** (N=346)	**No Maintenance*** (N=347)	**Maintenance*** (N=305)
		N (%)		
**Radiation**				
Full dose received[†]	319 (92)	320 (92)	323 (93)	278 (91)
Full dose no delay/reduction	274 (79)	267 (77)	285 (82)	223 (73)
Dose or fraction not reported	4(1)	1 (<1)	4(1)	1 (<1)
**Chemotherapy during chemoradiation****				
Completed both weeks as per protocol	270 (78) [§]	252 (73)	-	-
Any delay, dose reduction or both[¶]	71 (21)	92 (27)	-	-
Insufficient data	4 (1)	2 (<1)	-	-
**Chemotherapy during maintenance therapy**[€#]	-	-	**Prior MMC** (N=146)	**Prior CisP** (N=159)
Completed both courses as per protocol	-	-	68 (47)	72 (45)
Any delay, dose reduction or both[¶]	-	-	58 (40)	51 (32)
No chemotherapy	-	-	20 (14)	36 (23)

*39 patients randomised to MMC (n=20) or CisP (n=19) were not randomised to maintenance therapy.
[†]50.4 Gy in 28 fractions with or without interruptions
**2 patients randomised to CisP were given MMC. The reasons were low GFR post-randomisation (n=1), and administrative error (n=1). 2 patients were randomised to MMC but were given CisP during week 5 (one on clinician's advice and the other due to toxicity from 5FU during week 5).
[§]includes n=6 with confirmed overdose of MMC ranging from 22 to 27 mg/day
[¶]patients counted only once
[€]Those randomised to maintenance alone (n=344) included in the analysis
[#]56 patients did not receive maintenance therapy, 32 patient decision, 9 patient unwell, 3 clinical decision, 1 withdrew, 11 toxicity.

Table 4. Response according to age, gender, tumour size, tumour spread and N stage at randomisation among 691 patients who had tumour assessment data at all 3 time-points

Baseline factors	Response			
	Assessment 1		Assessment 3	
	cCR rate*	Absolute difference	cCR rate*	Difference
**Age, median**		0.3% (-7.8 to +8.4) P=0.94		-1.4% (-7.2 to +4.4) P=0.65
<65 years	63.9%		85.0%	
≥65 years	63.6%		86.4%	
**Gender**		5.8% (-1.8 to +13.3) P=0.13		7.7% (1.9 to 13.5) P=0.006
Female	65.9%		88.2%	
Male	60.2%		80.5%	
**Tumour size**		20.2% (12.1 to 28.2) P<0.001		9.9% (3.7 to 16.0) P<0.001
≤5 cm (T1+T2)	73.4%		89.7%	
>5 cm (T3)	53.2%		79.8%	
**Tumour spread****		18.1% (7.1 to 29.1) P<0.001		7.1 (-1.8 to +16.0 P=0.08
No spread	65.9%		86.0%	
Spread	47.8%		78.9%	
**N stage**		12.2% (4.3 to 20.0) P=0.002		10.1% (4.0 to 16.3) P<0.001
N0	67.6%		88.5%	
N+	55.4%		78.4%	

*Percentages based on patients with primary tumour response data at all three time-points (N=691)

**Tumour spread = T4 i.e. spread to neighbouring organs.

Chi-squared tests used

Table 5. Sensitivity analysis based on imputation of missing data for nodal status; Cox regression analyses (691 patients who had tumour assessment data at all 3 time-points)

Assessments	cCR (N, %)	HR (95% C) (cCR vs not-cCR)			
		PFS		OS	
		Crude	Adjusted*	Crude	Adjusted*
**Missing nodal status coded as node negative**		**N=691**	**N=452**	**N=691**	**N=452**
1	482 (70%)	0.60 (0.45 to 0.80) P<0.001	0.65 (0.44 to 0.94) P=0.02	0.53 (0.37 to 0.75) P<0.001	0.77 (0.49 to 1.22) P=0.27
2	585 (85%)	0.36 (0.26 to 0.49) P<0.001	0.42 (0.27 to 0.64) P<0.001	0.28 (0.20 to 0.41) P<0.001	0.42 (0.25 to 0.69) P=0.001
3	603 (87%)	0.15 (0.11 to 0.21) P<0.001	0.16 (0.10 to 0.24) P<0.001	0.17 (0.12 to 0.24) P<0.001	0.23 (0.14 to 0.39) P<0.001
**Missing nodal status coded as node positive**		**N=691**	**N=452**	**N=691**	**N=452**
1	426 (62%)	0.71 (0.54 to 0.95) P=0.02	0.85 (0.59 to 1.24) P=0.41	0.70 (0.49 to 0.99) P=0.04	1.06 (0.67 to 1.67) P=0.81
2	526 (76%)	0.52 (0.38 to 0.71) P<0.001	0.59 (0.40 to 0.88) P=0.01	0.48 (0.34 to 0.70) P<0.001	0.59 (0.37 to 0.89) P=0.03
3	584 (85%)	0.21 (0.15 to 0.28) P<0.001	0.20 (0.13 to 0.31) P<0.001	0.22 (0.15 to 0.31) P<0.001	0.31 (0.19 to 0.51) P<0.001
**Missing nodal status coded as missing**					
1	426 (62%)	**N=635** 0.62 (0.46 to 0.84) P=0.002	**N=417** 0.71 (0.48 to 1.05) P=0.09	**N=635** 0.57 (0.40 to 0.81) P=0.002	**N=417** 0.87 (0.55 to 1.40) P=0.57
2	526 (76%)	**N=632** 0.36 (0.30 to 0.50) P<0.001	**N=417** 0.43 (0.30 to 0.66) P<0.001	**N=632** 0.30 (0.21 to 0.44) P<0.001	**N=417** 0.42 (0.25 to 0.70) P=0.001
3	584 (85%)	**N=672** 0.15 (0.11 to 0.21) P<0.001	**N=441** 0.16 (0.11 to 0.25) P<0.001	**N=672** 0.17 (0.12 to 0.25) P<0.001	**N=441** 0.24 (0.14 to 0.41) P=0.001

P-values are from Wald test.

*Adjusted for age, gender, site of primary, tumour differentiation, histology, baseline WBC, baseline platelets, baseline haemoglobin, tumour size, N stage and treatment.

Table 6. Sensitivity and false positive rates (FPR) of three assessments with regard to overall survival endpoint at 1 and 3 years (n=940)

	Year 1			Year 3		
	Sensitivity (%)	FPR (%)	Accuracy (%)	Sensitivity (%)	FPR (%)	Accuracy (%)
**cCR Vs being alive**	Alive at year 1	Died before year 1		Alive at year 3	Died before year 3	
Assessment 1	68.3 (479/701)	50.0 (13/26)	67.7	70.2 (442/630)	51.6 (50/97)	67.3
Assessment 2	84.8 (656/774)	32.1 (19/28)	84.2	87.4 (604/691)	55.0 (61/111)	81.6
Assessment 3	87.6 (726/829)	20.0 (4/16)	87.4	91.3 (678/743)	49.1 (52/106)	86.2
**Not-cCR Vs risk of death**	Died before year 1	Alive at year 1		Died before year 3	Alive at year 3	
Assessment 1	50.6 (13/26)	31.7 (222/701)	67.7	48.5 (47/97)	29.8 (188/630)	67.3
Assessment 2	67.9 (19/28)	15.3 (118/774)	84.2	45.1 (50/111)	12.6 (87/691)	81.6
Assessment 3	80.0 (16/20)	12.4 (103/829)	87.4	50.9 (54/106)	8.8 (65/743)	86.2

cCR *vs* being alive: Sensitivity = number cCR at a certain assessment and alive at 1 or 3 years / total alive at 1 or 3 years.
FPR= number cCR at a certain assessment and died by 1 or 3 years/total died before 1 or 3 years

not-cCR *vs* death: Sensitivity = number not-cCR at a certain assessment and died by 1 or 3 years/total died before 1 or 3 years
FPR = number not-cCR at a certain assessment and alive at 1 or 3 years/total alive at 1 or 3 years

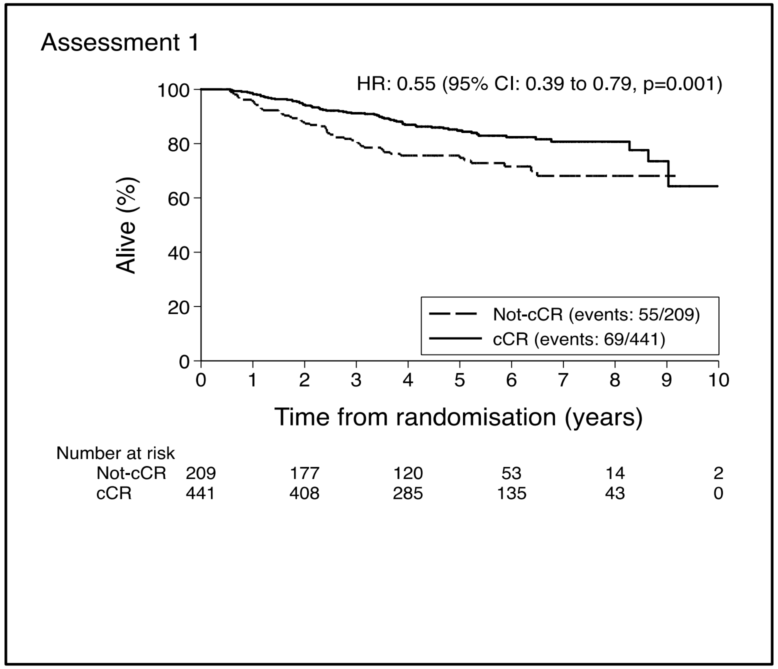

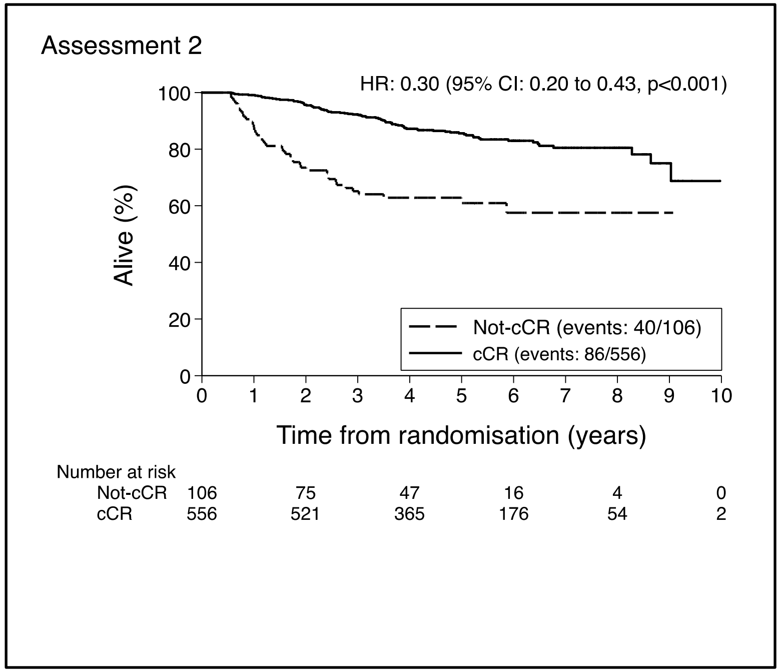

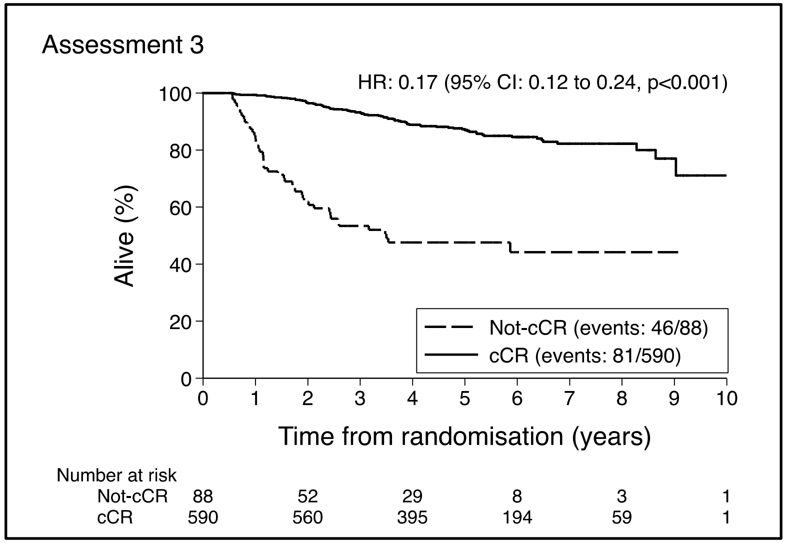

Figure 1. Overall survival according to response at Assessments 1, 2 and 3 (4 weeks post-CRT and 18 and 26 weeks from the start of CRT respectively) among 691 patients who had tumour assessments at all 3 time-points. The 5-year OS rates for cCR and not-cCR groups were - Assessment 1: 85% and 75%; Assessment 2: 86% and 61%; Assessment 3: 87% and 48%.

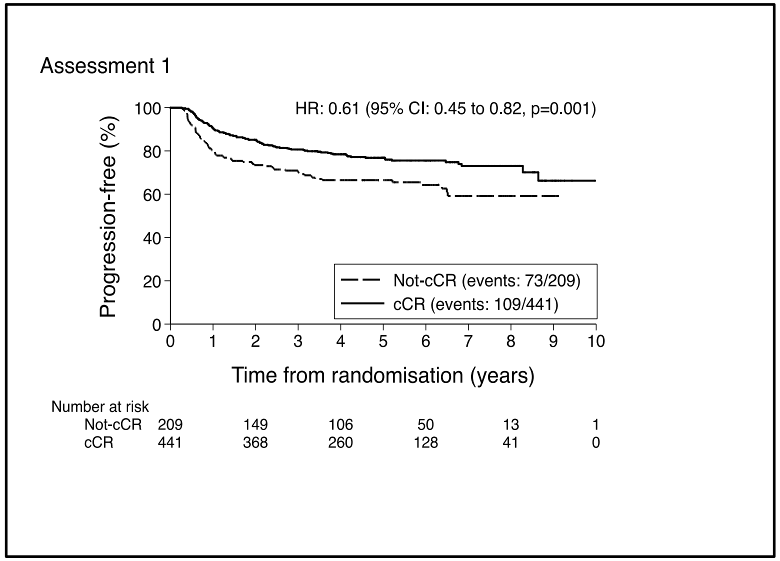

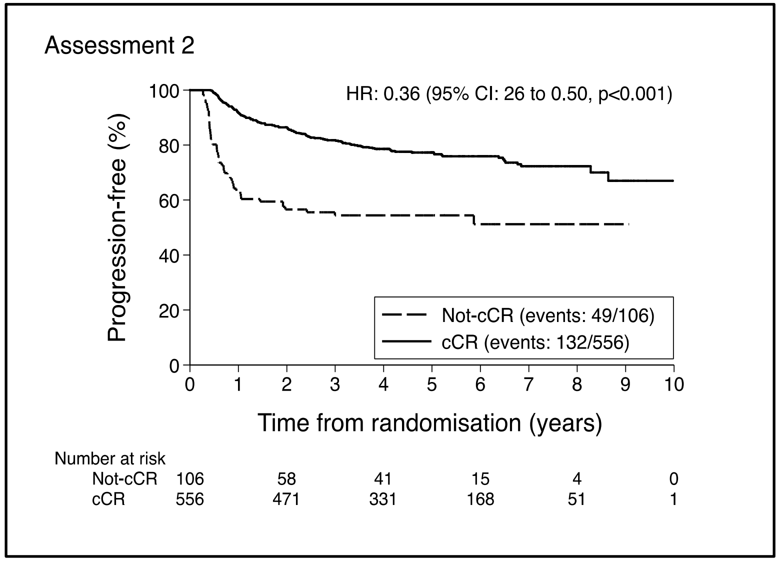

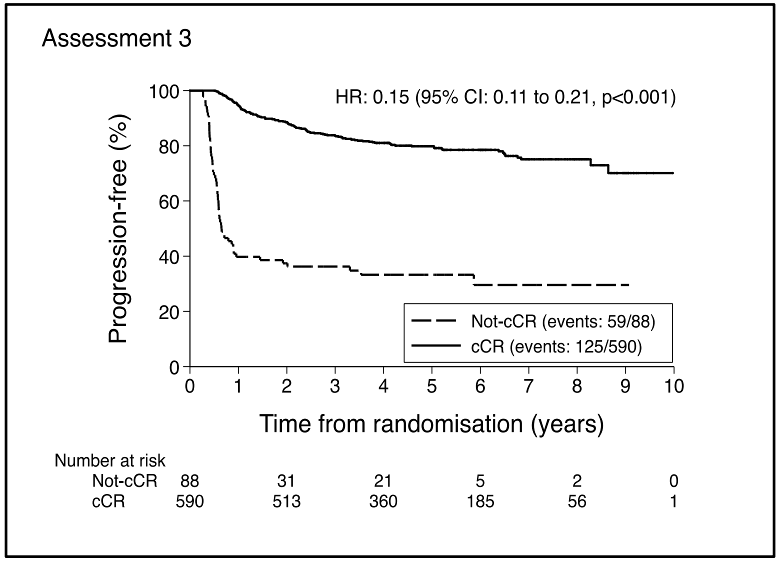

Figure 2. Progression-free survival according to response at Assessments 1, 2 and 3 (4 weeks post-CRT and 18 and 26 weeks from the start of CRT respectively) among 691 patients who had tumour assessments at all 3 time-points. The 5-year PFS rates for cCR and not-cCR groups were - Assessment 1: 77% and 67%; Assessment 2: 77% and 54%; Assessment 3: 80% and 33%. The apparent flat curves early on is due to the first assessment of disease being at 11 weeks post-randomisation (the first PFS event was at 3.3 months).

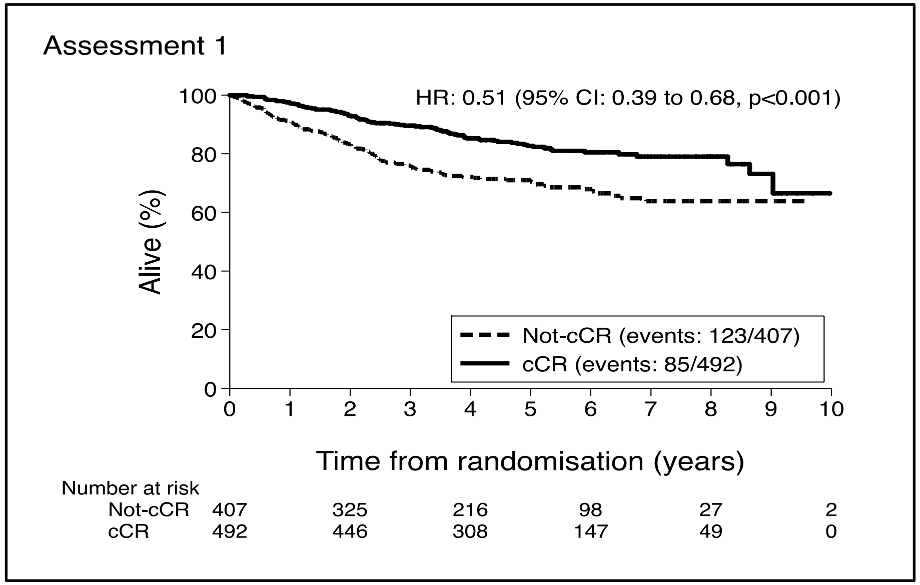

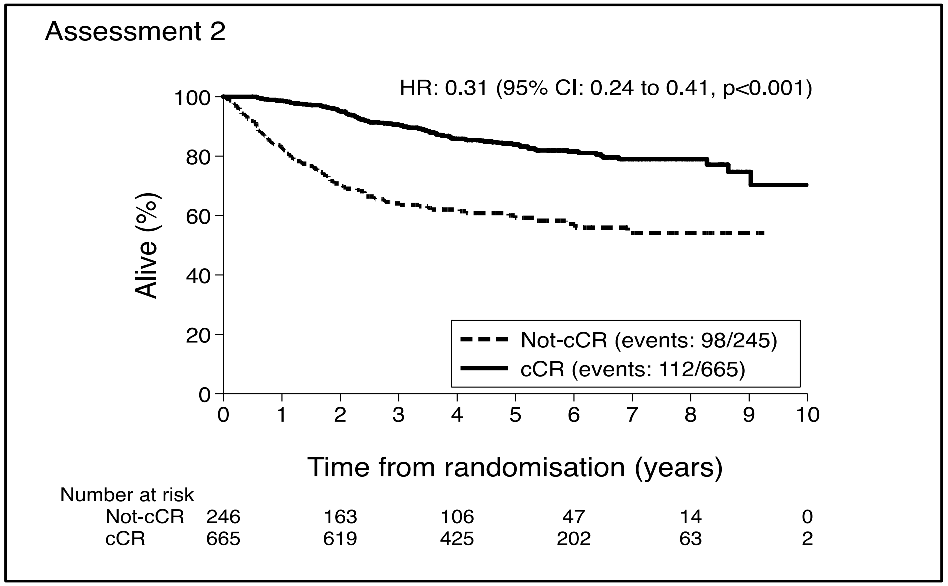

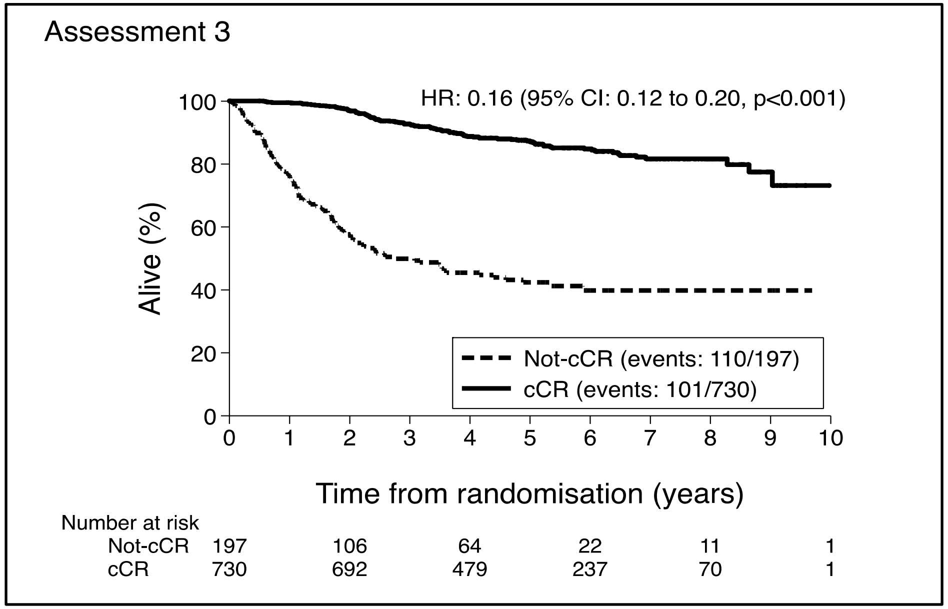

Figure 3. Overall survival among all 940 patients were included in the analysis, and where the cCR status was unknown, it was assumed to be not-cCR.

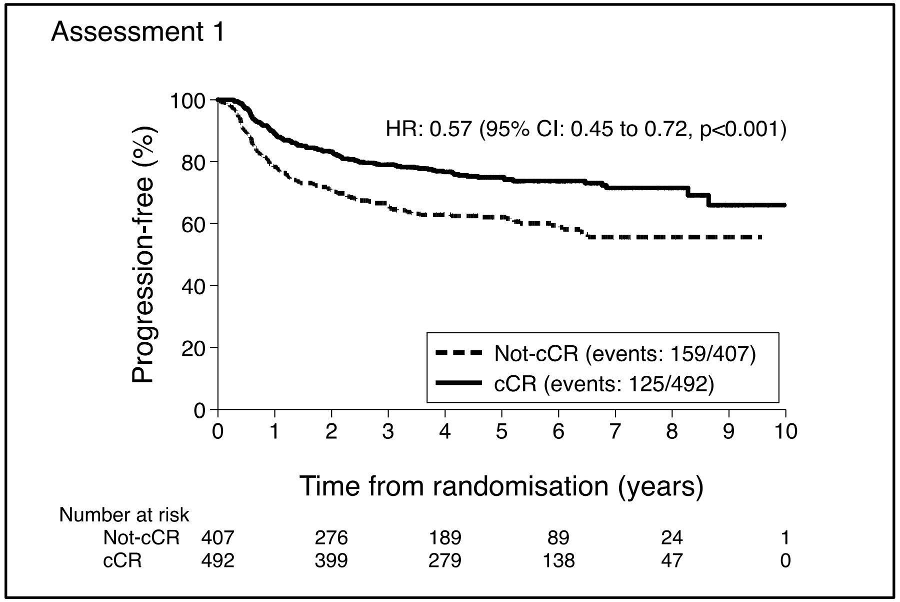

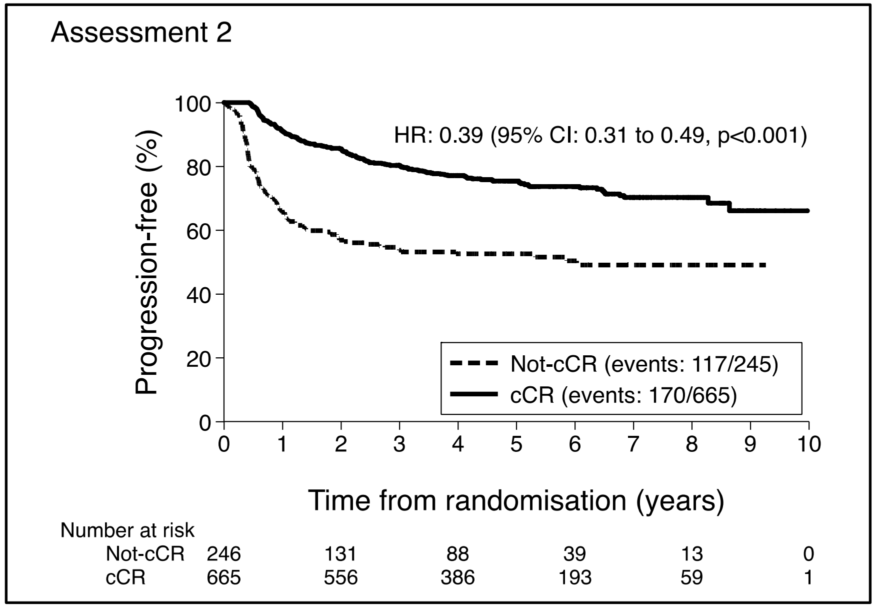

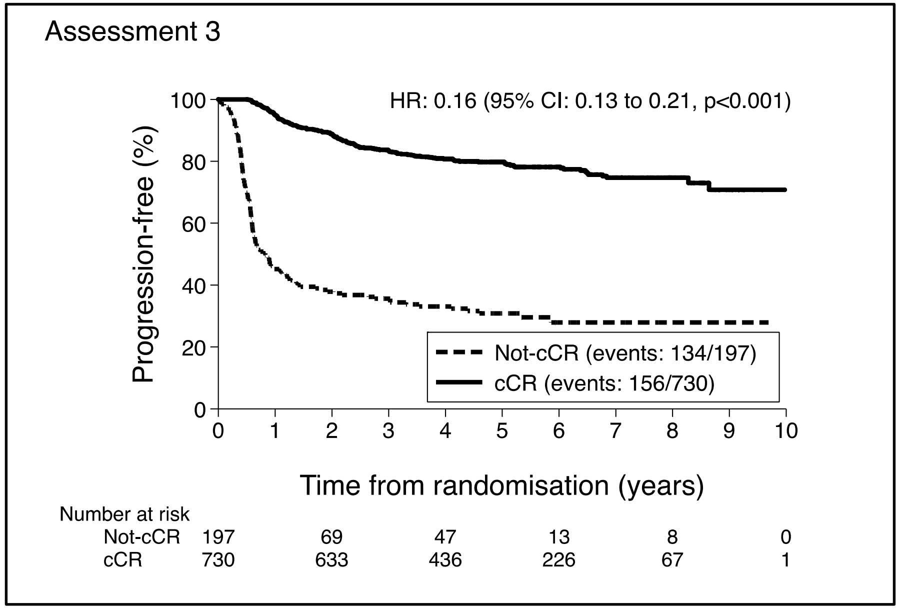

Figure 4. Progression-free survival among all 940 patients were included in the analysis, and where the cCR status was unknown, it was assumed to be not-cCR.

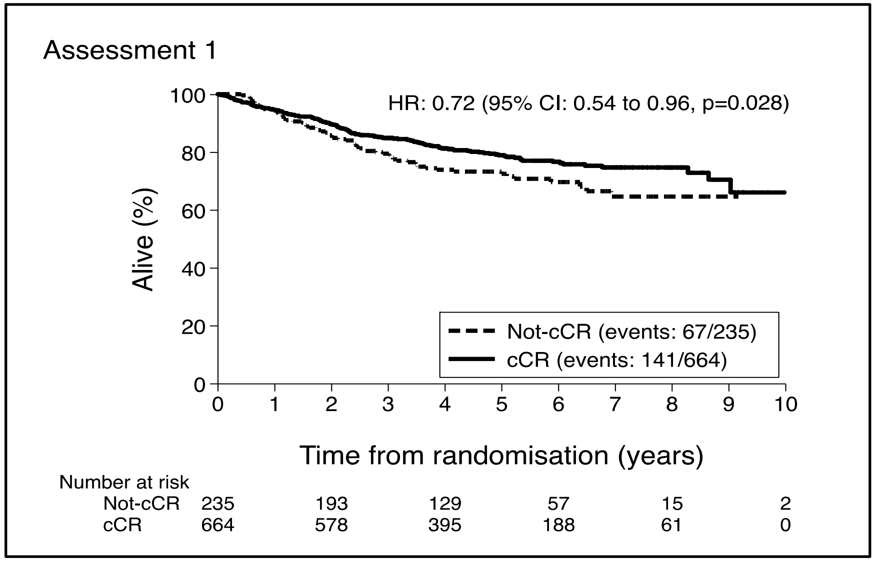

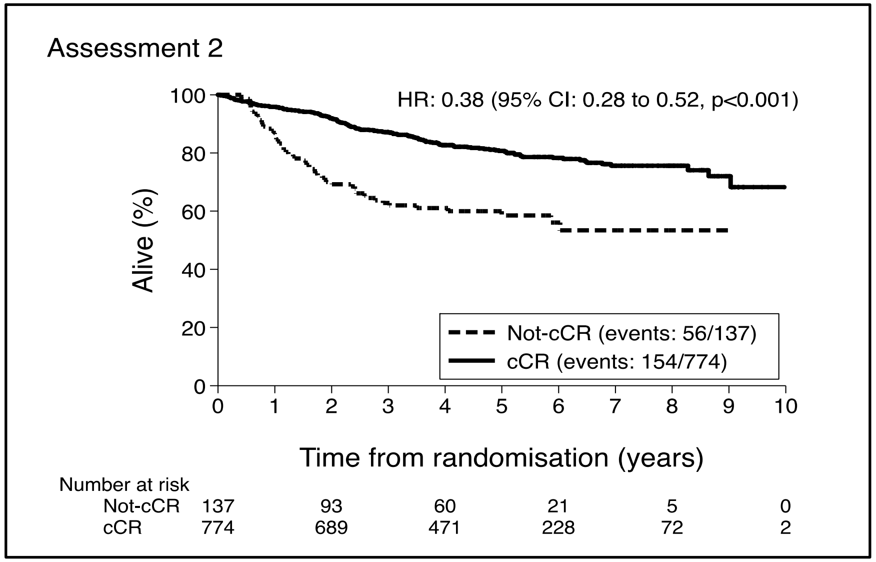

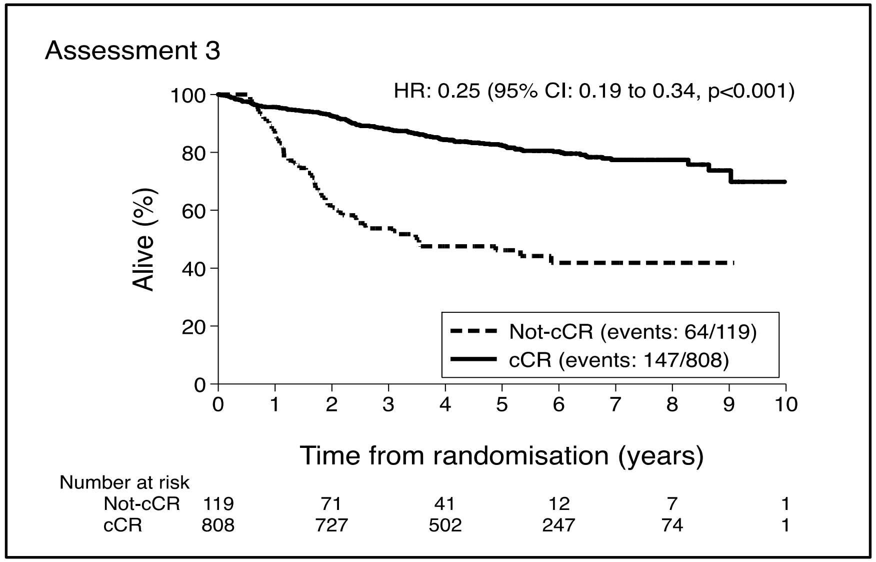

Figure 5. Overall survival among all 940 patients were included in the analysis, and where the cCR status was unknown, it was assumed to be cCR.

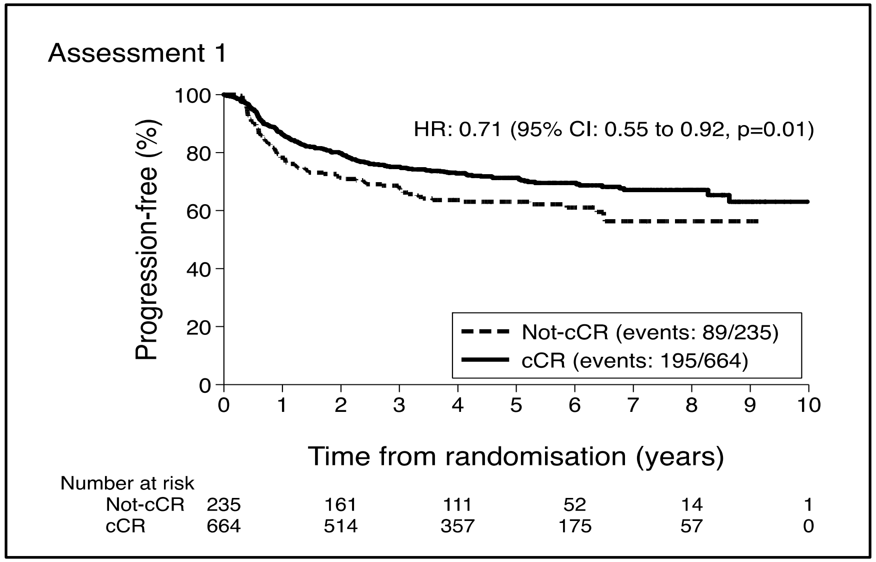

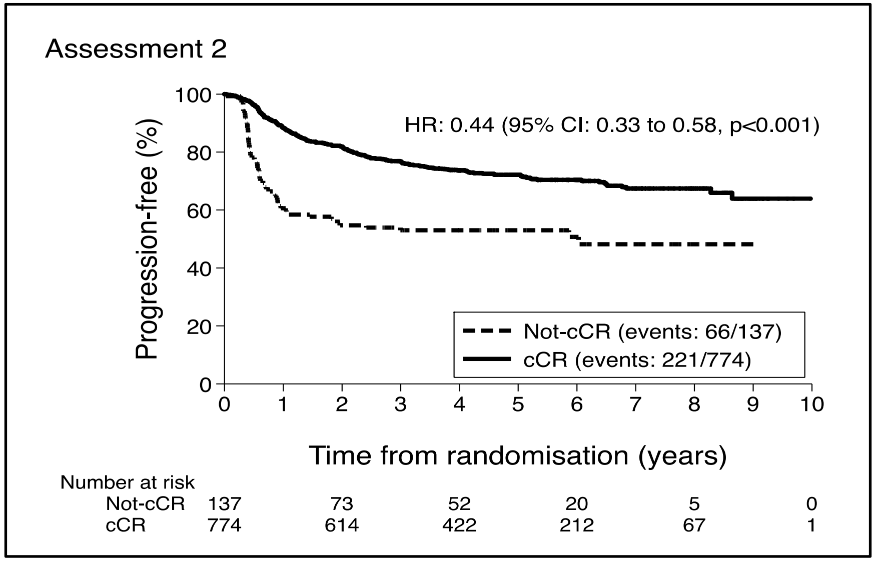

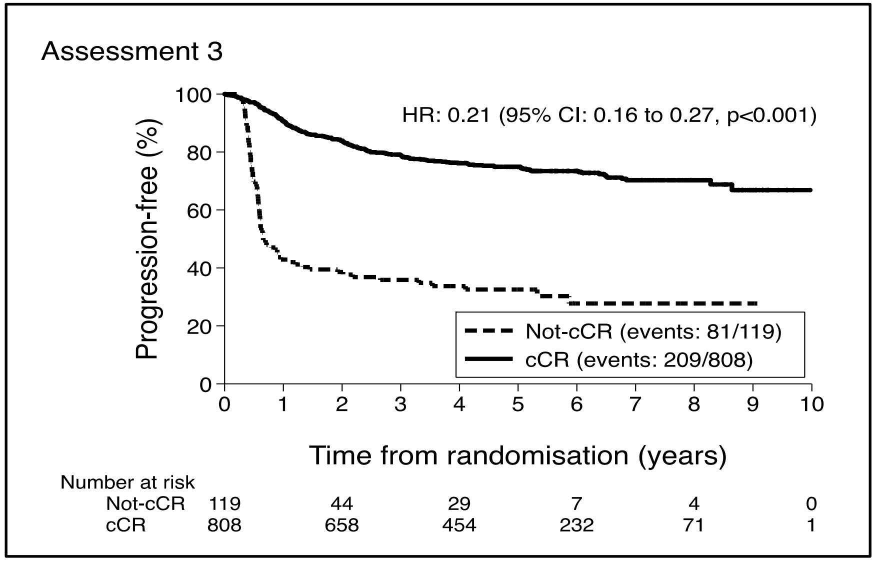

Figure 6. Progression-free survival among all 940 patients were included in the analysis, and where the cCR status was unknown, it was assumed to be cCR.
